# Comparing the genomes of *Helicobacter pylori* clinical strain UM032 and Mice-adapted derivatives

**DOI:** 10.1186/1757-4749-5-25

**Published:** 2013-08-19

**Authors:** Yalda Khosravi, Vellaya Rehvathy, Wei Yee Wee, Susana Wang, Primo Baybayan, Siddarth Singh, Meredith Ashby, Junxian Ong, Arlaine Anne Amoyo, Shih Wee Seow, Siew Woh Choo, Tim Perkins, Eng Guan Chua, Alfred Tay, Barry James Marshall, Mun Fai Loke, Khean Lee Goh, Sven Pettersson, Jamuna Vadivelu

**Affiliations:** 1 Department of Medical Microbiology, University of Malaya, Kuala Lumpur, Malaysia; 2 Dental Research and Training Unit, Faculty of Dentistry, University of Malaya, Kuala Lumpur, Malaysia; 3 Pacific Biosciences, Menlo Park, California, USA; 4 PacBio Singapore, Singapore, Singapore; 5 National Cancer Centre, Singapore, Singapore; 6 School of Pathology and Laboratory Medicine, Faculty of Medicine, Dentistry and Health Sciences, University of Western Australia, Perth, Western Australia, Australia; 7 The Marshall Centre for Infectious Diseases Research and Training, University of Western Australia, Perth, Western Australia, Australia; 8 Department of Medicine, University of Malaya, Kuala Lumpur, Malaysia; 9 Department of Microbiology, Tumor and Cell Biology (MTC), Karolinska Institutet, Stockholm, Sweden; 10 School of Biological Sciences, Nanyang Technological University, Singapore, Singapore

**Keywords:** *Helicobacter pylori*, PacBio Single Molecule, Real-Time (SMRT) technology, Clinical *H. pylori*, Mice-adapted

## Abstract

**Background:**

*Helicobacter pylori* is a Gram-negative bacterium that persistently infects the human stomach inducing chronic inflammation. The exact mechanisms of pathogenesis are still not completely understood. Although not a natural host for *H. pylori*, mouse infection models play an important role in establishing the immunology and pathogenicity of *H. pylori*. In this study, for the first time, the genome sequences of clinical *H. pylori* strain UM032 and mice-adapted derivatives, 298 and 299, were sequenced using the PacBio Single Molecule, Real-Time (SMRT) technology.

**Result:**

Here, we described the single contig which was achieved for UM032 (1,599,441 bp), 298 (1,604,216 bp) and 299 (1,601,149 bp). Preliminary analysis suggested that methylation of *H. pylori* genome through its restriction modification system may be determinative of its host specificity and adaptation.

**Conclusion:**

Availability of these genomic sequences will aid in enhancing our current level of understanding the host specificity of *H. pylori*.

## Background

*Helicobacter pylori* persistently colonizes the human stomach to cause chronic gastritis, peptic ulcer disease, gastric adenocarcinoma, and gastric mucosa-associated lymphoid tissue (MALT) lymphoma [1]. The mechanisms involved in the pathogenesis of *H. pylori* infections are still not fully established [2]. Thus, experimental animal models that mimic human diseases are essential to provide information on etiopathogeny, immunity and therapy, as well as to improve our understanding on ways *H. pylori* can induce a diverse range of gastric pathologies [3,4]. Among various animal models available, mouse remains the most readily used animal model for studying *H. pylori*-induced diseases and have played important roles in the elucidation of factors required for colonization, distribution and persistence of infection [2].

## Methods

### Mice adaptation study

Adopting a similar strategy as in previous studies [2,5], a pool consisting of twelve clinical strains of *H. pylori* isolated from patients presenting for gastroscopy at the University of Malaya Medical Centre (UMMC) was inoculated intragastrically into five 4–6 weeks old male C57BL/6 mice. Multiple colonies of *H. pylori* were successfully recovered from the gastric tissue sample of a mouse (1/5) following necropsy two weeks post-infection. Random amplification of polymorphic DNA (RAPD) fingerprinting was used to trace back the mice-adapted isolates to its parental clinical strain, UM032. *H. pylori* UM032 was isolated from a patient presenting with peptic ulcer disease. Mice-adapted isolates from the first mouse passage were designated as 298 and were used for the second round of mouse passage to access the stability and infectivity of this mice-adapted strain. All three mice inoculated with the mice-adapted 298 strain were successfully infected and *H. pylori* isolated from the second passage were designed as 299. The animal study was performed with the approval of the SingHealth Institutional Research Committees (SHS IBC) and the Ethical Committee for Animal Research (Form No. SHS-IBC-201, January 2010).

### Genome sequencing

In this study, *H. pylori* DNA was isolated using the RTP Bacteria DNA Mini Kit (Invitek GmbH, Berlin, Germany). The extracted DNA samples were sequenced using Pacific Biosciences RS sequencing technology (Pacific Biosciences, Menlo Park, CA), yielding >20× average genome coverage. Each sample was prepared as a 10-kb insert library using C2 chemistry and sequenced on 8 Single-Molecule Real-Time (SMRT) cells.

### Assembly and annotation

D*e novo* assembly of the read sequences was created using the continuous long reads (CLR) following the Hierarchical Genome Assembly Process (HGAP) workflow (http://pacbiodevnet.com/) as available in SMRT Analysis v2.0. The genomes were annotated with the NCBI (National Center for Biotechnology Information) Prokaryotic Genomes Automatic Annotation Pipeline and NMPDR (National Microbial Data Resource) Rapid Annotation using Subsystem Technology (RAST) [6]. The SEED-Viewer was used to visualize the genome annotation and comparison generated by RAST [7].

### Submission of genome sequence

The genome sequence of the Helicobacter pylori strains UM032, 298 and 299 are available in DDBJ/EMBL/GenBank under Accession numbers CP005490, CP006610 and CP005491 respectively.

## Quality assurance

The genomic DNA was isolated from pure bacterial isolate (positive for urease, catalase and oxidase tests) and was further confirmed with *16SrRNA* sequencing and genotyping of bacterial virulence factors. Bioinformatic assessment of potential contamination of the genomic library by allochthonous microorganisms was done using PGAAP and RAST annotation systems.

## Initial findings

### Genome characteristics

**Figure 1:**
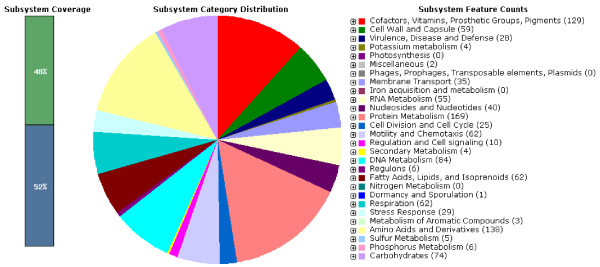
**Subsystem distribution statistic of *Helicobacter pylori *strain UM032 based on genome annotation performed according to RAST server.**

**Figure 2:**
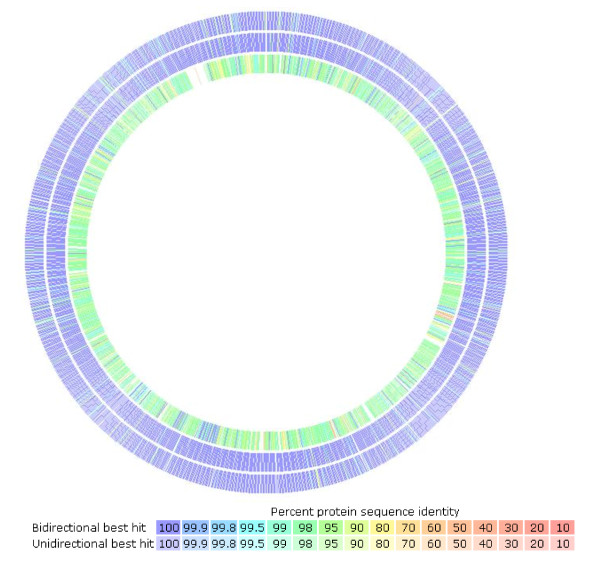
**Genome sequence comparison of *Helicobacter pylori *298 (outer) 299 (middle) and Shi470 (inner) when aligned with reference genome, UM032, using RAST program.** Intensity of color indicates degree of protein identity (legend).

**Figure 3:**
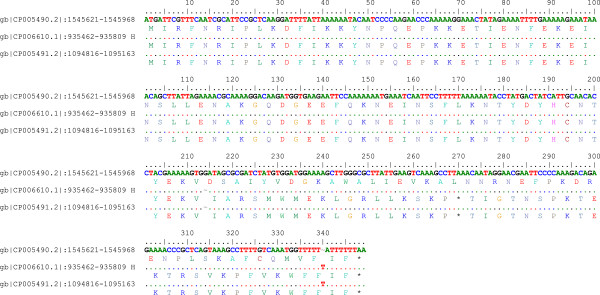
**Pair-wise alignment of putative type IIS restriction modification enzyme.** Deletion of single guanine nucleotide at position 214 resulted in downstream frame-shift mutation and prematured termination of the RM enzymes encoded by 298 and 299.

Based on the assembled genomes with HGAP using PacBio long reads from a single library preparation, single contigs were achieved for UM032 (1,599,441 bp), 298 (1,604,216 bp) and 299 (1,601,149 bp). The GC content for all three assembled genomes was 38.8%. Additional information is included in the sequencing reports: UM032 (Additional file 1), 298 (Additional file 2) and 299 (Additional file 3). Figure 1 describes the subsystem distribution of the parental clinical strain, UM032. Figure 2 shows the sequence homology between 298, 299 and Shi470 with reference to UM032. *H. pylori* Shi470 was predicted to be among those closest to UM032 with score of 470. Neither gene lost nor gain was found in the mice-adapted derivatives (298 and 299) when compared to the parental strain (UM032). Interestingly, total base modifications detected through the PacBio RS sequencing platform as described under Table 1was reducing with passaging in mice. In addition, a 348 b.p. gene encoding for a putative type IIS restriction modification (R-M) enzyme in *H. pylori* UM032 was found in to truncated in 298 and 299 (Figure 3). Thus, methylation may be a mean of host adaptation by *H. pylori* and may have an important role in determining host specificity.

**Table 1:** Type of base modifications and associated motifs detected

**Motif**	**Modification type**	** # of motifs detected**		
		**UM032**	**298**	**299**
G**A**NTC	m6A	5,393	5,428	5,397
CC**A**TC	m6A	2,257	2,261	2,258
G**A**GG	m6A	4,585	4,598	4,580
TCNG**A**	m6A	2,531	2,544	2,534
G**A**TC	m6A	10,195	10,210	10,175
**C**CGG	m4C	3,414	3,424	3,420
TGC**A**	m6A	11,221	11,199	11,185
CY**A**NNNNNNNTRG	m6A	2,303	2,319	2,305
ATTA**A**T	m6A	865	865	865
A**C**NGT	m4C	1,077	1,056	1,056
C**A**TG	m6A	13,446	13,339	13,361
GAA**A**G	Unknown	4,332	4,839	4,851
Others		59,182	43,338	36,469
**Total**		**120,801**	**105,420**	**98,456**

The availability of complete sequences of mice-adapted strains and their parental clinical isolate will provide important information that contributes towards our understanding of the host specific and adaptation of *H. pylori*. In addition, it will help in extrapolate results obtained using mice model to the natural human host of *H. pylori*. *H. pylori* 298 strain will be used for *H. pylori* colonizing studies in mice.

### Putative gene clusters responsible for survival and virulence of *H. pylori*

**Figure 4:**
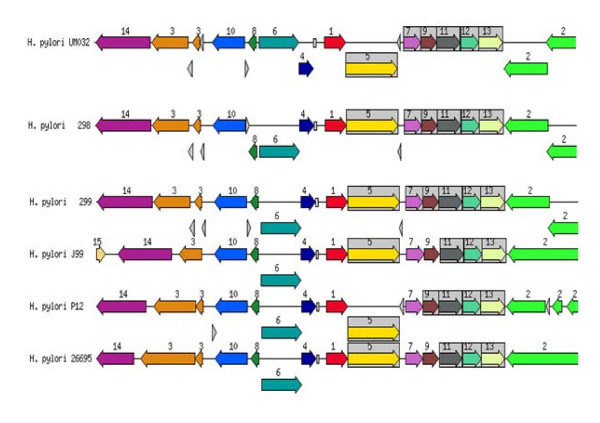
**Genetic relatedness of urease gene cluster with closely related bacteria.** 1: urease beta subunit/urease gama subunit, 2: cell division protein *Ftsk*, 3: outer membrane protein, 4: lipoprotein signal peptidase, 5: urease alpha subunit, 6: phosphoglucosamine mutase, 7: urea channel *ure*I, 8: SSU ribosomal protein S20P, 9: urease accessory protein *ure*E, 10: peptide chain release factor I, 11: urease accessory protein *ure*F, 12: urease accessory protein *ure*G, 13: urease accessory protein *ure*D, 14: dentin sialophosphoprotein preproprotein.

**Figure 5:**
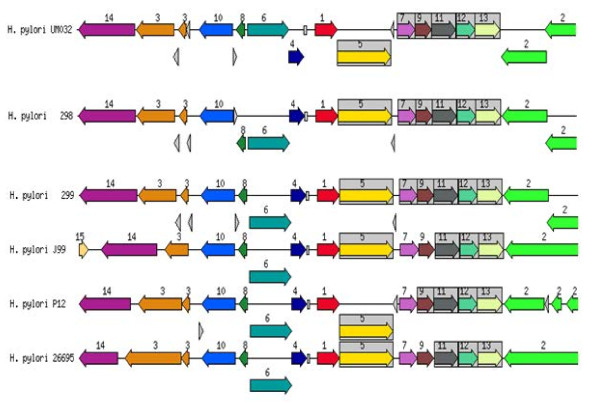
**Genetic relatedness of *vac*A cluster with closely related bacteria.** 1: vacuolating cytotoxin, 2: hypothetical protein, 3: haemin uptake system ATP-binding protein, 4: cysteinyl-Trna-SYNTHETASE, 5: IRON III, 6: dehydrogenases with different specificities, 7: proposted peptidoglycan lipid, 8: hypothetical protein, 9: hypothetical protein, 10: DNA damage inducible protein J, 11: holliday junction DNA helicase RUUA, 12: putative outer membrane protein, 13: hypothetical protein.

*H. pylori* possess genes for cytosolic urease biosynthesis, which is governed by a seven-gene cluster, are essential for its survival in the acidic gastric environment [8]. *H. pylori* vacuolating cytotoxin A (*Vac*A) is an important virulence factor of the bacterium [9]. Using the SEED database, genetic relatedness of the urease gene cluster and *vac*A for the clinical strain (UM032) and its mice-adapted counterparts (298 and 299) in comparison to other known *H. pylori* strains are shown in Figures 4 and 5.

## Future directions

To our knowledge, this is the first genome sequence of *H. pylori* isolated from human and mouse using PacBio SMRT Technology. Comparative genomic and more-detailed methylomic analysis of these data is in process and will be included in future publications. Mice-adapted *H. pylori* described here will be used in future *H. pylori* infection studies in mice.

## Availability of supporting data

The data sets supporting the results of this article are included within the additional files.

## Competing interests

The authors declare that they have no competing interests.

## Authors’ contributions

YK, MFL, JV, SSW and SP planned the experiments. YK and MFL prepared the *H. pylori* for inoculation, isolated the bacteria from mice, performed genetic fingerprinting and extracted the DNA. JO, AAA and SWS were responsible for animal infection and study. SW, PB, SS and MA carried out the genomic sequencing and initial bioinformatics. VR, TP, ENG, AT carried out further bioinformatics analysis. BJM, JV, KLG, SP and MFL are senior co-authors. All authors have read the manuscript and approved.

## Supplementary Material

Additional file 1 Assembly report for UM032. Click here for file

Additional file 2 Assembly report for 298. Click here for file

Additional file 3 Assembly report for 299. Click here for file
