# Correction: Comparing the genomes of *Helicobacter pylori* clinical strain UM032 and mice-adapted derivatives

**DOI:** 10.1186/1757-4749-6-11

**Published:** 2014-05-08

**Authors:** Yalda Khosravi, Vellaya Rehvathy, Wei Yee Wee, Susana Wang, Primo Baybayan, Siddarth Singh, Meredith Ashby, Junxian Ong, Arlaine Anne Amoyo, Shih Wee Seow, Siew Woh Choo, Tim Perkins, Eng Guan Chua, Alfred Tay, Barry James Marshall, Mun Fai Loke, Khean Lee Goh, Sven Pettersson, Jamuna Vadivelu

**Affiliations:** 1 Department of Medical Microbiology, University of Malaya, Kuala Lumpur, Malaysia; 2 Dental Research and Training Unit, Faculty of Dentistry, University of Malaya, Kuala Lumpur, Malaysia; 3 Pacific Biosciences, Menlo Park, California, USA; 4 PacBio Singapore, Singapore, Singapore; 5 National Cancer Centre, Singapore, Singapore; 6 School of Pathology and Laboratory Medicine, Faculty of Medicine, Dentistry and Health Sciences, University of Western Australia, Perth, Western Australia, Australia; 7 The Marshall Centre for Infectious Diseases Research and Training, University of Western Australia, Perth, Western Australia, Australia; 8 Department of Medicine, University of Malaya, Kuala Lumpur, Malaysia; 9 Department of Microbiology, Tumor and Cell Biology (MTC), Karolinska Institutet, Stockholm, Sweden; 10 School of Biological Sciences, Nanyang Technological University, Singapore, Singapore

## Correction

After publication of this work [1], we discovered that due to an inadvertent mistake, Figures 4 and 5 in the original article were essentially the same figure. Amendment to Figure 5 (Figure 1 here) has now been added. We offer our sincerest apologies for this oversight.

**Figure 1:**
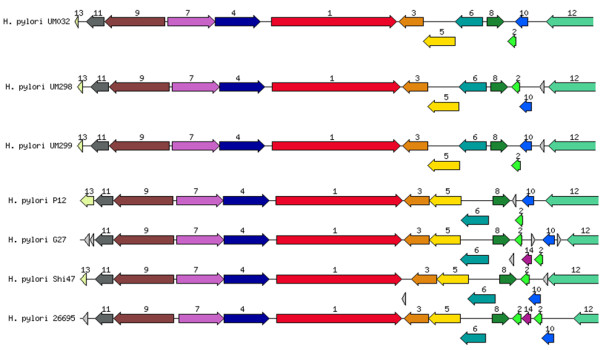
**Genetic relatedness of *vac*A cluster with closely related bacteria.** 1: vacuolating cytotoxin, 2: hypothetical protein, 3: haemin uptake system ATP-binding protein, 4: cysteinyl-Trna-SYNTHETASE, 5: IRON III, 6: dehydrogenases with different specificities, 7: proposted peptidoglycan lipid, 8: hypothetical protein, 9: hypothetical protein, 10: DNA damage inducible protein J, 11: holliday junction DNA helicase RUUA, 12: putative outer membrane protein, 13: hypothetical protein.
