# Supplementary material for: Comparing the genomes of Helicobacter pylori clinical strain UM032 and Mice-adapted derivatives
Source: Gut Pathog. 2013 Aug 19;5:25. doi: 10.1186/1757-4749-5-25 (PMC3751790; doi:10.1186/1757-4749-5-25)
Supplement: Additional file 1 — Assembly report for UM032. [file 1757-4749-5-25-S1.pdf]

Reports for Job hp32

SMRT Cells: 8 Movies: 8

Overview

Job Metric	Value
Adapter Dimers (%)	0.39
Short Inserts (%)	0.09
Post-Filter Polymerase Read Bases	567089444
Post-Filter Polymerase Reads	214554
Post-Filter Polymerase Read Length	2643
Post-Filter Polymerase Read Quality	0.857
Polished Contigs	1
N50 Contig Length	1599441
Sum of Contig Lengths	1599441
Mapped Reads	184857
Mapped Read Length of Insert	2084
Reference Length - scf7180000000002	1601272
Bases Called (%) - scf7180000000002	100.00
Consensus Concordance - scf7180000000002	99.8876
Coverage - scf7180000000002	269.19

Adapters

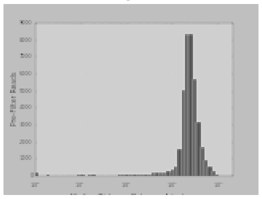

Observed Insert Length Distribution Histogram

Subread Filtering

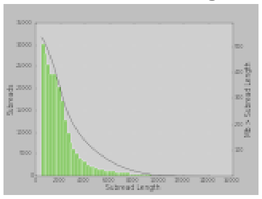

Subread Filtering

Coverage

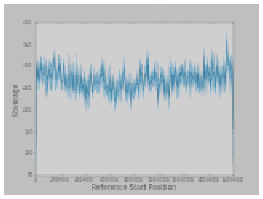

Coverage Across Reference

Coverage

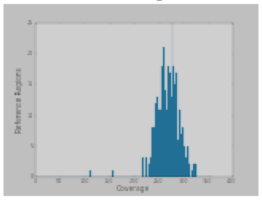

Depth of Coverage

Mapping

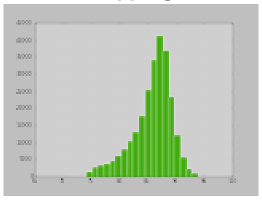

Mapped Subread Accuracy

Mapping

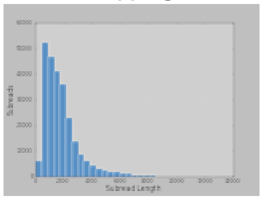

Mapped Subread Length

Mapping

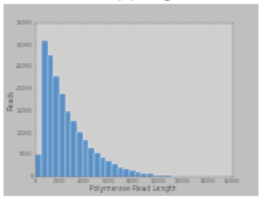

Mapped Polymerase Read Length

Corrections

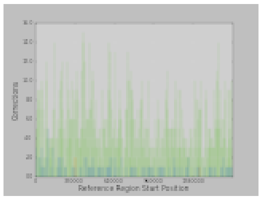

Corrections Across Contigs

Filtering

	Pre-Filter	Post-Filter
Polymerase Read Bases	737408468 bp	567089444 bp
Polymerase Reads	601224	214554
Polymerase Read Length	1227 bp	2643 bp
Polymerase Read Quality	0.386	0.857

Polymerase Read Length

Post-Filter

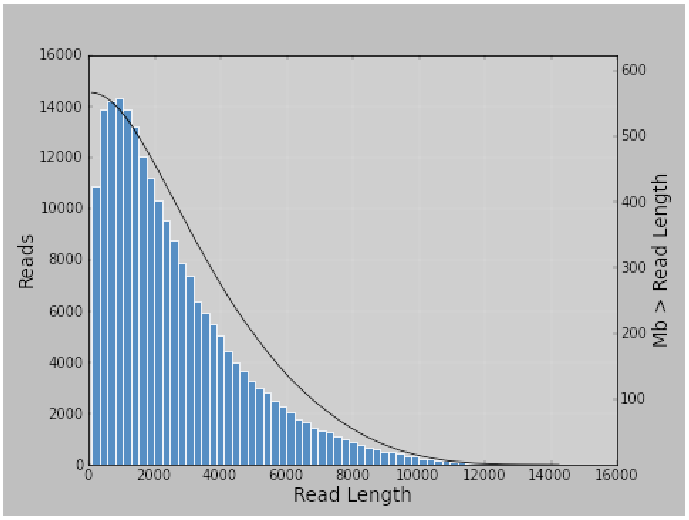

Polymerase Read Quality

Post-Filter

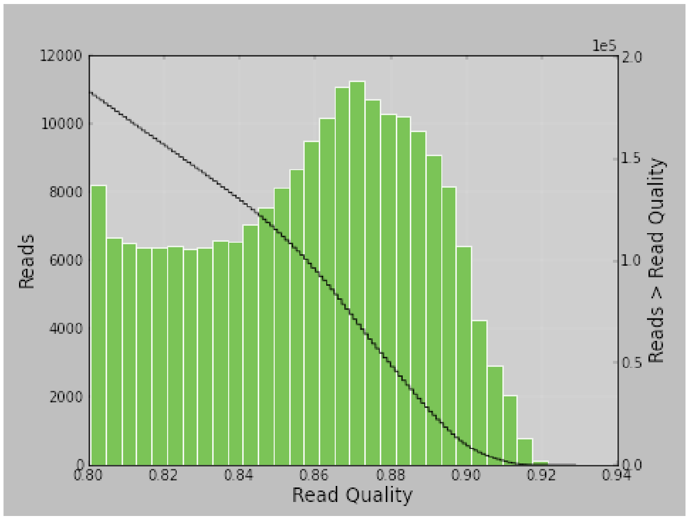

Subread Filtering

Subread Filtering

Post-Filter

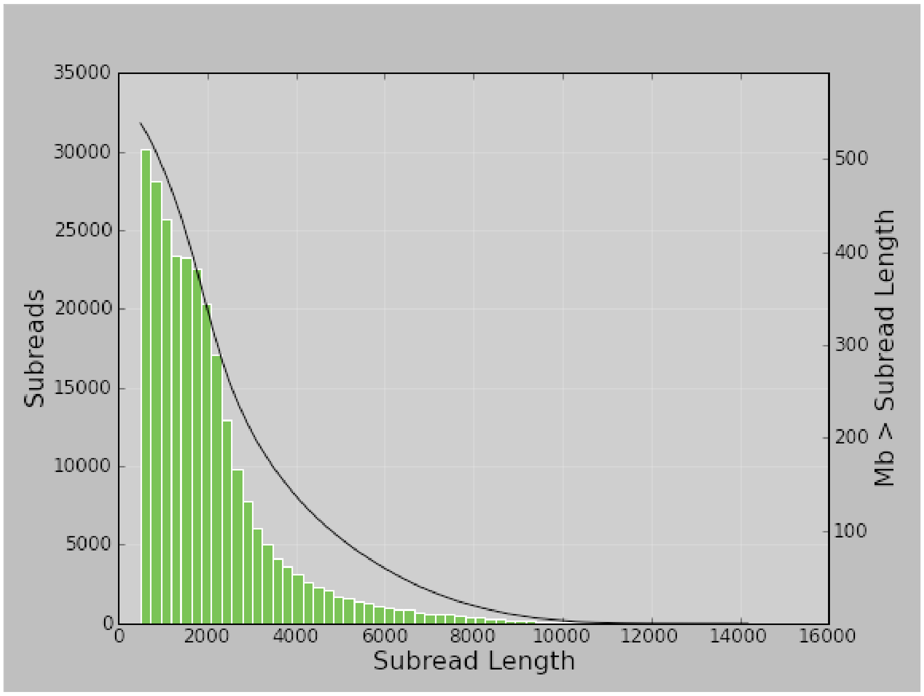

Adapters

Adapter Dimers (%)	0.39 %
Short Inserts (%)	0.09 %

Observed Insert Length Distribution Histogram

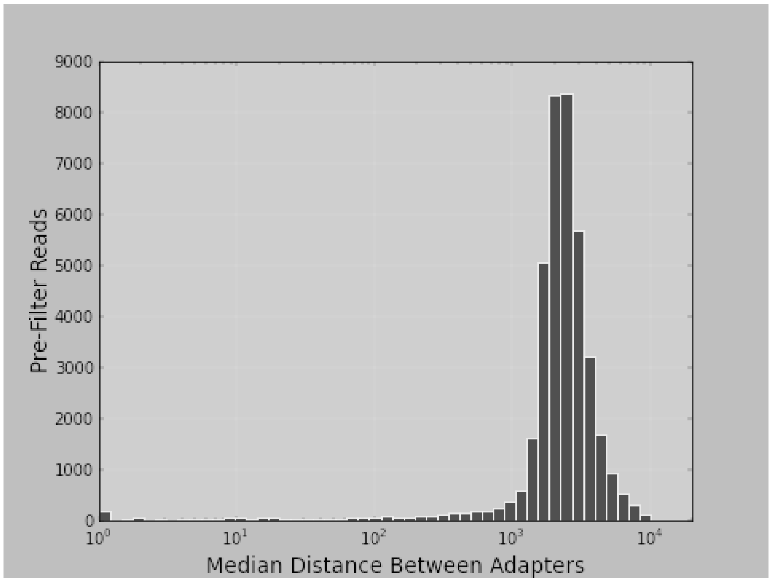

Loading

SMRT Cell ID	Productive ZMWs	Productivity 0 (%)	Productivity 1 (%)	Productivity 2 (%)
m120811_055117_42142_c100407420070000001523036611231251	75153	23.6	52.9	23.6
m120811_035516_42142_c100407420070000001523036611231250	75153	18.5	58.5	23.0
m120811_074621_42142_c100407420070000001523036611231252	75153	25.5	52.3	22.2
m120811_172446_42142_c100354232550000001523024909301255	75153	36.7	37.8	25.5
m120811_133139_42142_c100361060070000001523019709161212	75153	34.6	36.3	29.1
m120811_153010_42142_c100354232550000001523024909301254	75153	37.9	36.3	25.8
m120811_094153_42142_c100361060070000001523019709161210	75153	32.7	42.9	24.4
m120811_113638_42142_c100361060070000001523019709161211	75153	31.7	42.8	25.5

Pre-Assembly

Polymerase Read Bases	539606414	Length Cutoff	6322
Seed Bases	51001157	Pre-Assembled Bases	34805331
Pre-Assembled Yield	.682	Pre-Assembled Reads	6241
Pre-Assembled Read Length	5576	Pre-Assembled N50	6393

Polished Assembly

Polished Contigs	1	Max Contig Length	1599441
N50 Contig Length	1599441	Sum of Contig Lengths	1599441

Top Corrections

Sequence	Position	Correction	Type	Coverage	Confidence	Genotype
scf7180000000002	199706	199706_199707insT	INS	100	51	haploid
scf7180000000002	824949	824949delAC	DEL	100	51	haploid
scf7180000000002	1123564	1123564_1123565insT	INS	100	51	haploid
scf7180000000002	1310635	1310635_1310636insA	INS	100	51	haploid
scf7180000000002	1521609	1521609_1521610insA	INS	100	51	haploid
scf7180000000002	582	582_583insA	INS	15	50	haploid
scf7180000000002	21358	21358delA	DEL	100	50	haploid
scf7180000000002	23074	23074_23075insA	INS	100	50	haploid
scf7180000000002	49701	49701delA	DEL	100	50	haploid
scf7180000000002	54434	54434_54435insT	INS	100	50	haploid
scf7180000000002	56419	56419delT	DEL	100	50	haploid
scf7180000000002	63141	63141delA	DEL	100	50	haploid
scf7180000000002	82160	82160_82161insA	INS	100	50	haploid
scf7180000000002	102844	102844_102845insA	INS	100	50	haploid
scf7180000000002	123562	123562delT	DEL	100	50	haploid

scf7180000000002	142622	142622_142623insA	INS	100	50	haploid
scf7180000000002	147011	147011delA	DEL	100	50	haploid
scf7180000000002	161385	161385delCG	DEL	100	50	haploid
scf7180000000002	175288	175288delA	DEL	100	50	haploid
scf7180000000002	177938	177938delG	DEL	100	50	haploid
scf7180000000002	190753	190753_190754insA	INS	100	50	haploid
scf7180000000002	196962	196962delT	DEL	100	50	haploid
scf7180000000002	237461	237461delT	DEL	100	50	haploid
scf7180000000002	256983	256983delT	DEL	100	50	haploid
scf7180000000002	282782	282782delT	DEL	100	50	haploid
scf7180000000002	298509	298509delT	DEL	100	50	haploid
scf7180000000002	356906	356906_356907insT	INS	100	50	haploid
scf7180000000002	378962	378962delA	DEL	100	50	haploid
scf7180000000002	408643	408643delT	DEL	100	50	haploid
scf7180000000002	461247	461247_461248insA	INS	100	50	haploid
scf7180000000002	463716	463716_463717insA	INS	100	50	haploid
scf7180000000002	469643	469643_469644insT	INS	100	50	haploid
scf7180000000002	478782	478782delGC	DEL	100	50	haploid
scf7180000000002	483089	483089_483090insT	INS	100	50	haploid
scf7180000000002	506587	506587delC	DEL	100	50	haploid
scf7180000000002	521929	521929_521930insA	INS	100	50	haploid
scf7180000000002	533511	533511delA	DEL	100	50	haploid
scf7180000000002	554314	554314delA	DEL	100	50	haploid
scf7180000000002	564768	564768delA	DEL	100	50	haploid
scf7180000000002	576289	576289delA	DEL	100	50	haploid
scf7180000000002	595774	595774delA	DEL	100	50	haploid
scf7180000000002	612338	612338delT	DEL	100	50	haploid
scf7180000000002	623387	623387delT	DEL	100	50	haploid
scf7180000000002	626399	626399_626400insA	INS	100	50	haploid
scf7180000000002	653053	653053_653054insA	INS	100	50	haploid
scf7180000000002	696449	696449_696450insA	INS	100	50	haploid
scf7180000000002	748721	748721delA	DEL	100	50	haploid
scf7180000000002	748767	748767delG	DEL	100	50	haploid
scf7180000000002	761425	761425delT	DEL	100	50	haploid
scf7180000000002	865559	865559delT	DEL	100	50	haploid
scf7180000000002	866317	866317_866318insA	INS	100	50	haploid
scf7180000000002	904267	904267delT	DEL	100	50	haploid
scf7180000000002	919520	919520_919521insA	INS	100	50	haploid
scf7180000000002	938580	938580_938581insT	INS	100	50	haploid
scf7180000000002	957572	957572delA	DEL	100	50	haploid
scf7180000000002	1109013	1109013delA	DEL	100	50	haploid
scf7180000000002	1109812	1109812delA	DEL	100	50	haploid
scf7180000000002	1176044	1176044delA	DEL	100	50	haploid
scf7180000000002	1200505	1200505delT	DEL	100	50	haploid
scf7180000000002	1206161	1206161delA	DEL	100	50	haploid
scf7180000000002	1215931	1215931delT	DEL	100	50	haploid
scf7180000000002	1218266	1218266delG	DEL	100	50	haploid
scf7180000000002	1239389	1239389_1239390insT	INS	100	50	haploid
scf7180000000002	1329616	1329616delT	DEL	100	50	haploid
scf7180000000002	1345476	1345476delT	DEL	100	50	haploid
scf7180000000002	1468082	1468082delA	DEL	100	50	haploid
scf7180000000002	1474148	1474148delCA	DEL	100	50	haploid
scf7180000000002	1495368	1495368delT	DEL	100	50	haploid
scf7180000000002	1503914	1503914delTC	DEL	100	50	haploid
scf7180000000002	1518510	1518510delT	DEL	100	50	haploid
scf7180000000002	1580121	1580121delT	DEL	100	50	haploid
scf7180000000002	665	665_666insA	INS	21	49	haploid
scf7180000000002	966	966_967insC	INS	22	49	haploid
scf7180000000002	3854	3854delG	DEL	98	49	haploid
scf7180000000002	10599	10599delT	DEL	100	49	haploid
scf7180000000002	12612	12612delT	DEL	100	49	haploid
[illegible]	[illegible]	[illegible]	[illegible]	[illegible]	[illegible]	[illegible]

scf7180000000002	13102	13102delT	DEL	100	49	haploid
scf7180000000002	14909	14909delT	DEL	100	49	haploid
scf7180000000002	21911	21911delC	DEL	100	49	haploid
scf7180000000002	23281	23281_23282insT	INS	100	49	haploid
scf7180000000002	24724	24724delT	DEL	100	49	haploid
scf7180000000002	25921	25921delA	DEL	100	49	haploid
scf7180000000002	26496	26496delA	DEL	100	49	haploid
scf7180000000002	27829	27829delA	DEL	100	49	haploid
scf7180000000002	38634	38634_38635insA	INS	100	49	haploid
scf7180000000002	45647	45647delT	DEL	100	49	haploid
scf7180000000002	52595	52595delT	DEL	100	49	haploid
scf7180000000002	53148	53148delA	DEL	100	49	haploid
scf7180000000002	53216	53216_53217insT	INS	100	49	haploid
scf7180000000002	58037	58037delG	DEL	100	49	haploid
scf7180000000002	59926	59926delA	DEL	100	49	haploid
scf7180000000002	62472	62472delA	DEL	100	49	haploid
scf7180000000002	78599	78599delT	DEL	100	49	haploid
scf7180000000002	86708	86708delA	DEL	100	49	haploid
scf7180000000002	86719	86719delT	DEL	100	49	haploid
scf7180000000002	87871	87871_87872insA	INS	100	49	haploid
scf7180000000002	87962	87962delA	DEL	100	49	haploid
scf7180000000002	88370	88370delTT	DEL	100	49	haploid
scf7180000000002	89275	89275delT	DEL	100	49	haploid
scf7180000000002	92843	92843delT	DEL	100	49	haploid

Corrections

Reference	Reference Length	Bases Called (%)	Consensus Concordance	Coverage
scf7180000000002	1601272	100.00	99.8876	269.19

Corrections Across Contigs

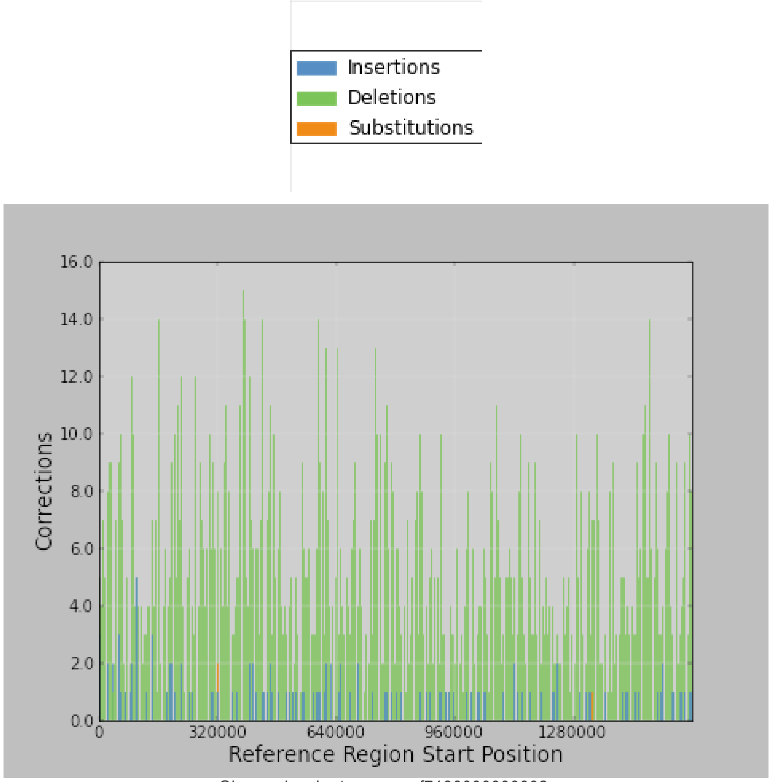

Observed variants across scf7180000000002.

Coverage

Coverage	269.19
Missing Bases (%)	0.00

Coverage Across Reference

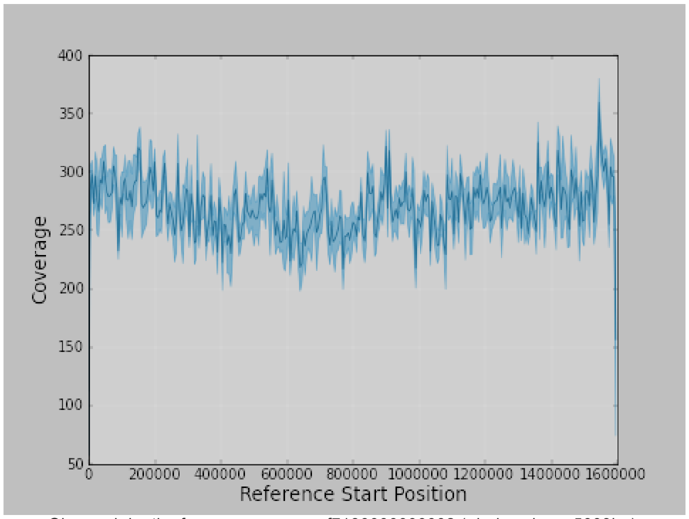

Observed depth of coverage across scf7180000000002 (window size = 5003bp).

Depth Of Coverage

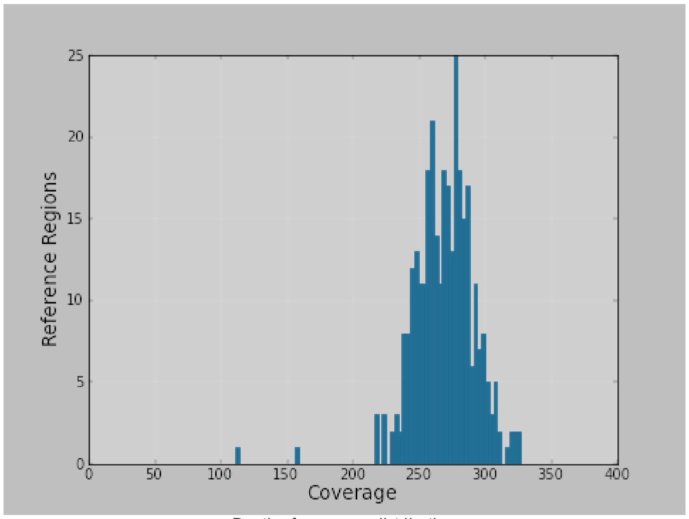

Depth of coverage distribution

Mapping

Post-Filter Reads	214554	Mapped Polymerase Read Length 95%	6788 bp
Mapped Read Length of Insert	2084 bp	Mapped Polymerase Read Length Max	14239 bp

	Mapped Reads	Mapped Polymerase Read Length	Mapped Subreads	Mapped Subread Bases	Mapped Subread Length	Mapped Subread Accuracy
All Movies	184857	2655 bp	254556	474259085 bp	1863 bp	85.69%
c100407420070000001523036611231251_s1_p0	27908	2897 bp	40447	77686343	1920 bp	85.58%
c100354232550000001523024909301254_s1_p0	15976	2759 bp	21722	43084337	1983 bp	85.77%
c100354232550000001523024909301255_s1_p0	17201	2619 bp	23131	43870628	1896 bp	85.71%
c100361060070000001523019709161210_s1_p0	22644	2398 bp	29965	52630285	1756 bp	85.79%
c100407420070000001523036611231250_s1_p0	31380	2878 bp	45172	86640754	1918 bp	85.66%
c100361060070000001523019709161211_s1_p0	23017	2320 bp	29705	51655770	1738 bp	85.65%
c100361060070000001523019709161212_s1_p0	19316	2305 bp	24736	43258009	1748 bp	85.79%
c100407420070000001523036611231252_s1_p0	27415	2855 bp	39678	75432959	1901 bp	85.66%

Mapped Subread Accuracy

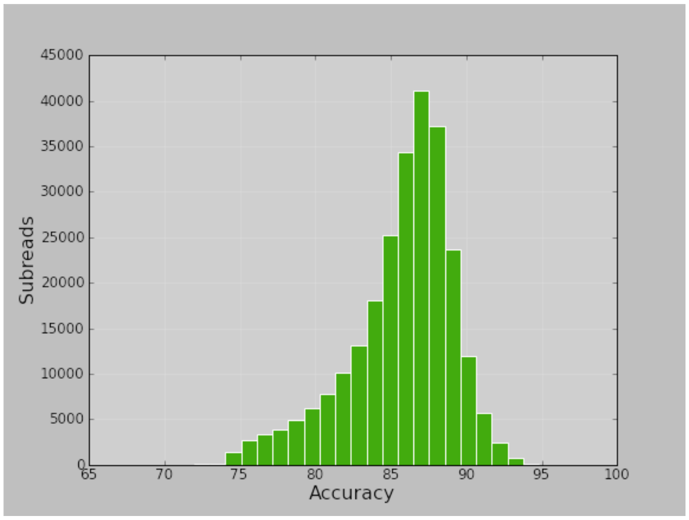

Mapped Subread Length

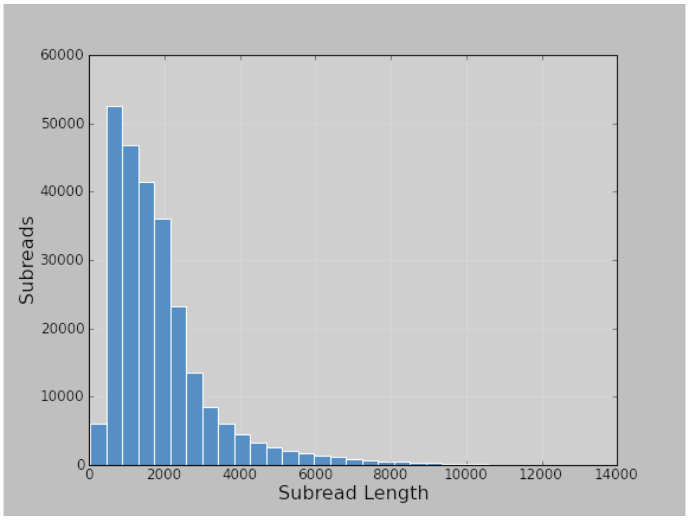

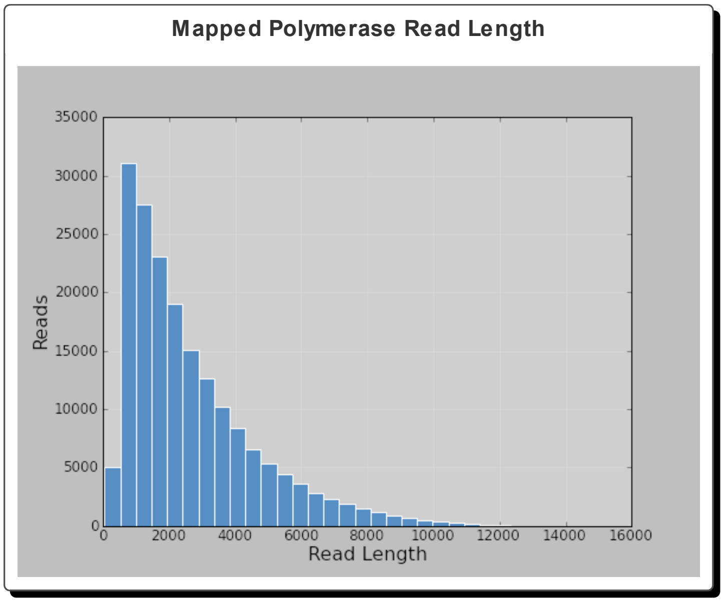

Generated by SMRT® Portal. Tue Jun 04 21:11:35 PDT 2013
For Research Use Only. Not for use in diagnostic procedures.
