# Supplementary material for: Comparing the genomes of Helicobacter pylori clinical strain UM032 and Mice-adapted derivatives
Source: Gut Pathog. 2013 Aug 19;5:25. doi: 10.1186/1757-4749-5-25 (PMC3751790; doi:10.1186/1757-4749-5-25)
Supplement: Additional file 2 — Assembly report for 298. [file 1757-4749-5-25-S2.pdf]

Reports for Job Hp 298

SMRT Cells: 7 Movies: 7

Overview

Job Metric	Value
Adapter Dimers (%)	11.76
Short Inserts (%)	1.82
Post-Filter Polymerase Read Bases	676430063
Post-Filter Polymerase Reads	169114
Post-Filter Polymerase Read Length	4000
Post-Filter Polymerase Read Quality	0.842
Polished Contigs	1
N50 Contig Length	1604216
Sum of Contig Lengths	1604216
Mapped Reads	121138
Mapped Read Length of Insert	3036
Reference Length - scf7180000000002	1604151
Bases Called (%) - scf7180000000002	100.00
Consensus Concordance - scf7180000000002	99.9981
Coverage - scf7180000000002	245.45

Adapters

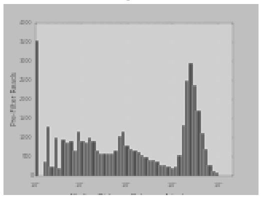

Observed Insert Length Distribution Histogram

Subread Filtering

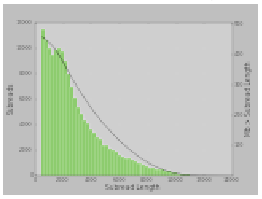

Subread Filtering

Coverage

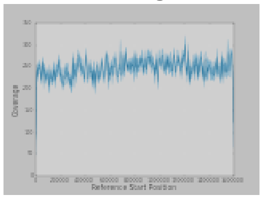

Coverage Across Reference

Coverage

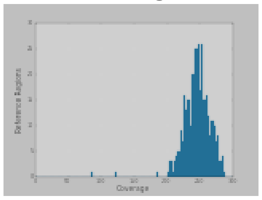

Depth of Coverage

Mapping

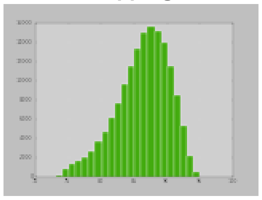

Mapped Subread Accuracy

Mapping

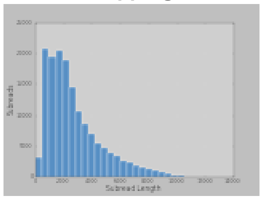

Mapped Subread Length

Mapping

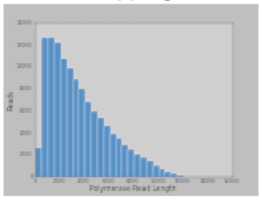

Mapped Polymerase Read Length

Corrections

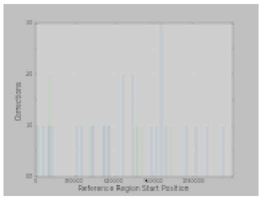

Corrections Across Contigs

Filtering

	Pre-Filter	Post-Filter
Polymerase Read Bases	955840704 bp	676430063 bp
Polymerase Reads	526071	169114
Polymerase Read Length	1817 bp	4000 bp
Polymerase Read Quality	0.389	0.842

Polymerase Read Length

Post-Filter

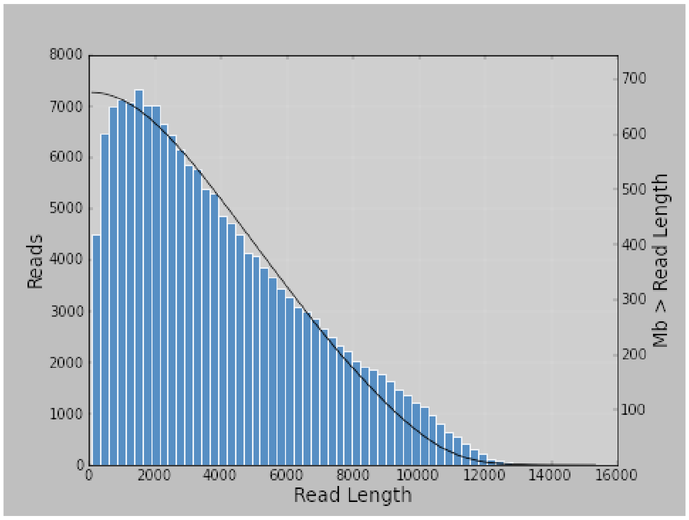

Polymerase Read Quality

Post-Filter

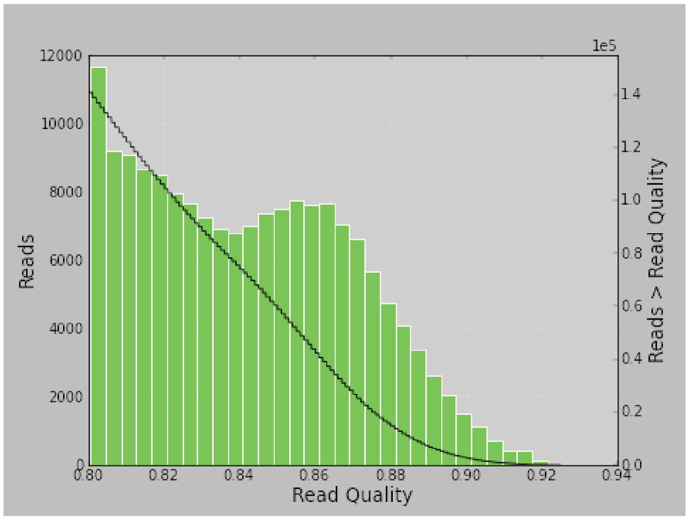

Subread Filtering

Subread Filtering

Post-Filter

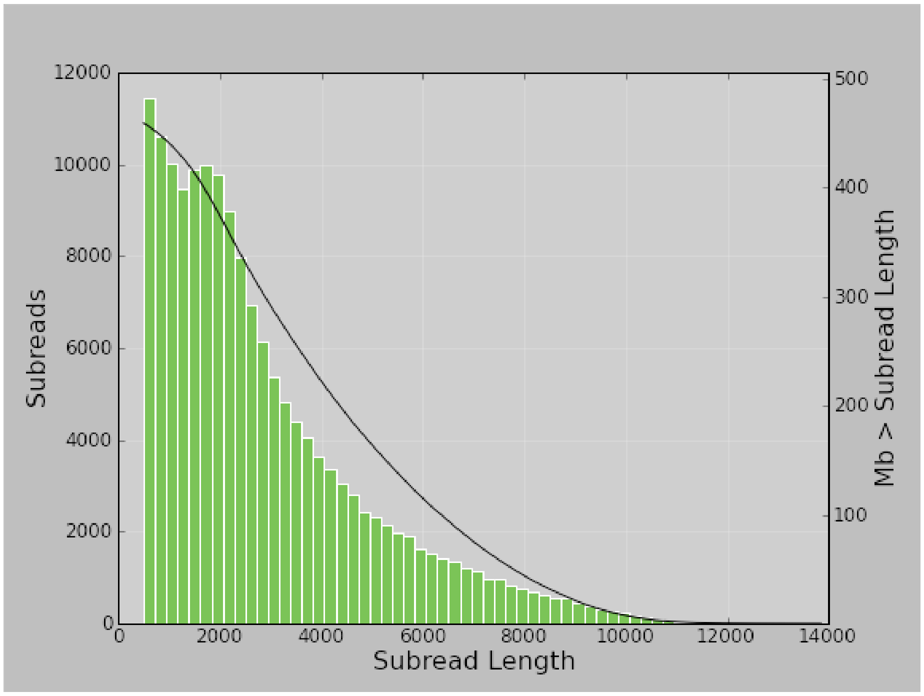

Adapters

Adapter Dimers (%)	11.76 %
Short Inserts (%)	1.82 %

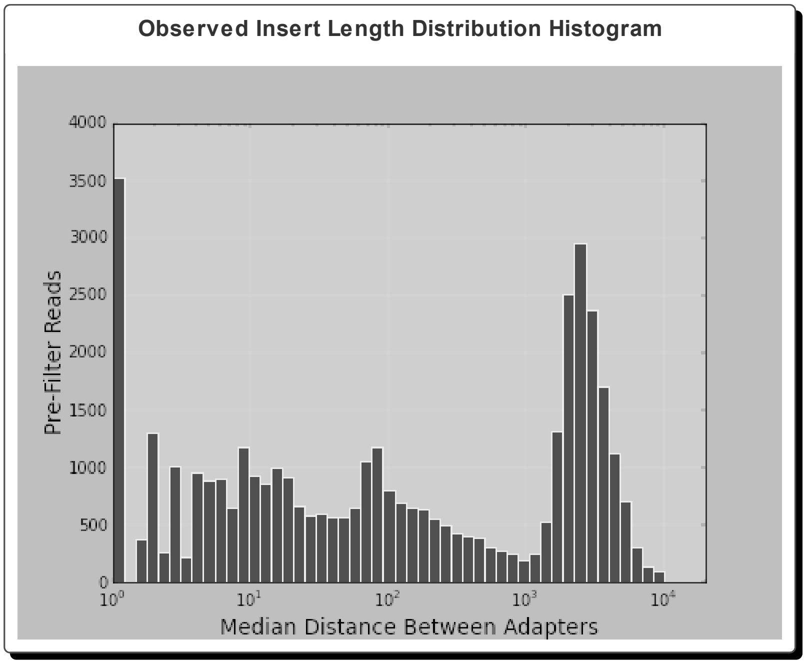

Loading

SMRT Cell ID	Productive ZMWs	Productivity 0 (%)	Productivity 1 (%)	Productivity 2 (%)
m120901_052216_42142_c100436120630000001523043602011382	75153	3.9	39.7	56.4
m120901_071006_42142_c100436120630000001523043602011383	75153	3.2	49.6	47.2
m120901_085756_42142_c100436120630000001523043602011384	75153	2.2	48.0	49.8
m120901_142126_42142_c100432341270000001523045902011321	75153	2.6	47.5	49.9
m120901_104546_42142_c100436120630000001523043602011385	75153	3.5	45.5	50.9
m120901_033426_42142_c100436120630000001523043602011381	75153	2.8	42.7	54.5
m120901_123336_42142_c100432341270000001523045902011320	75153	2.7	48.7	48.6

Pre-Assembly

Polymerase Read Bases	460069830	Length Cutoff	7753
Seed Bases	51015073	Pre-Assembled Bases	38914626
Pre-Assembled Yield	.762	Pre-Assembled Reads	5516
Pre-Assembled Read Length	7054	Pre-Assembled N50	7860

Polished Assembly

Polished Contigs	1	Max Contig Length	1604216
N50 Contig Length	1604216	Sum of Contig Lengths	1604216

Top Corrections

Sequence	Position	Correction	Type	Coverage	Confidence	Genotype
scf7180000000002	102859	102859_102860insG	INS	100	50	haploid
scf7180000000002	592255	592255_592256insG	INS	100	50	haploid
scf7180000000002	705424	705424_705425insCC	INS	100	50	haploid
scf7180000000002	1233355	1233355_1233356insG	INS	100	50	haploid
scf7180000000002	18804	18804_18805insG	INS	100	49	haploid
scf7180000000002	68680	68680_68681insC	INS	100	49	haploid
scf7180000000002	330422	330422_330423insC	INS	100	49	haploid
scf7180000000002	375065	375065_375066insC	INS	100	49	haploid
scf7180000000002	438608	438608delA	DEL	100	49	haploid
scf7180000000002	472830	472830_472831insT	INS	100	49	haploid
scf7180000000002	550575	550575_550576insC	INS	100	49	haploid
scf7180000000002	559682	559682_559683insC	INS	100	49	haploid
scf7180000000002	595711	595711_595712insC	INS	100	49	haploid
scf7180000000002	816271	816271delT	DEL	100	49	haploid
scf7180000000002	941362	941362_941363insC	INS	100	49	haploid
scf7180000000002	987192	987192_987193insG	INS	100	49	haploid
scf7180000000002	1057138	1057138_1057139insC	INS	100	49	haploid
scf7180000000002	1097010	1097010delT	DEL	100	49	haploid
scf7180000000002	1392236	1392236_1392237insG	INS	100	49	haploid
scf7180000000002	1528331	1528331_1528332insG	INS	100	49	haploid
scf7180000000002	22206	22206delA	DEL	100	48	haploid
scf7180000000002	110261	110261_110262insG	INS	100	48	haploid
scf7180000000002	113011	113011delG	DEL	100	48	haploid
scf7180000000002	142254	142254_142255insC	INS	100	48	haploid
scf7180000000002	460188	460188_460189insC	INS	100	48	haploid
scf7180000000002	787457	787457_787458insGG	INS	100	48	haploid
scf7180000000002	830937	830937_830938insC	INS	100	48	haploid
scf7180000000002	869942	869942delC	DEL	100	48	haploid
scf7180000000002	1300773	1300773_1300774insG	INS	100	48	haploid
scf7180000000002	111607	111607delA	DEL	100	47	haploid
scf7180000000002	1026657	1026657_1026658insCCC	INS	100	47	haploid

Corrections

Reference	Reference Length	Bases Called (%)	Consensus Concordance	Coverage
scf7180000000002	1604151	100.00	99.9981	245.45

Corrections Across Contigs

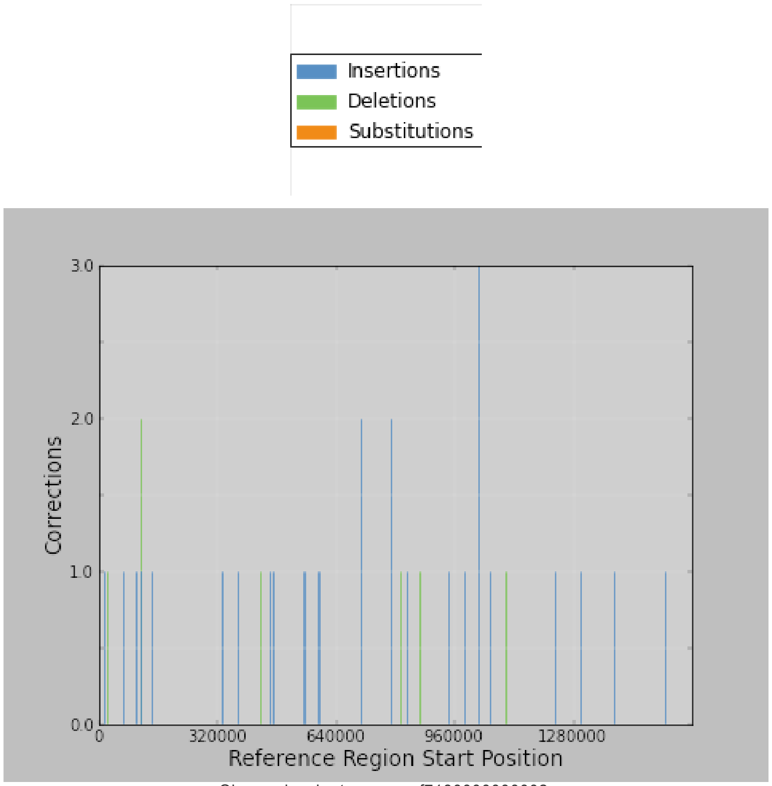

Observed variants across scf7180000000002.

Coverage

Coverage	245.45
Missing Bases (%)	0.00

Coverage Across Reference

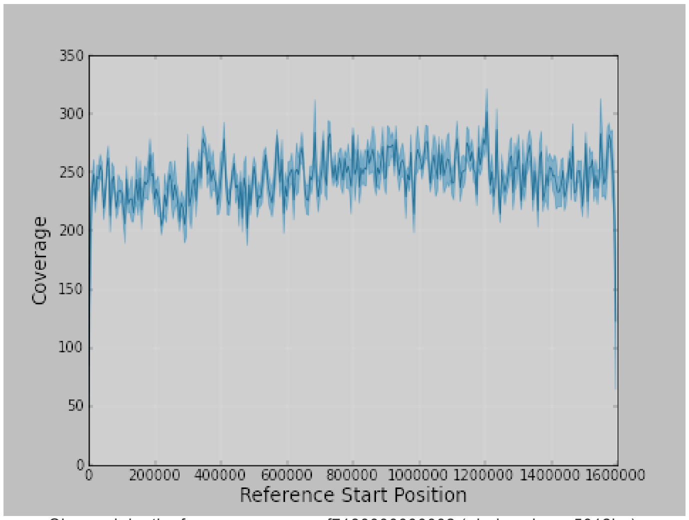

Observed depth of coverage across scf7180000000002 (window size = 5012bp).

Depth Of Coverage

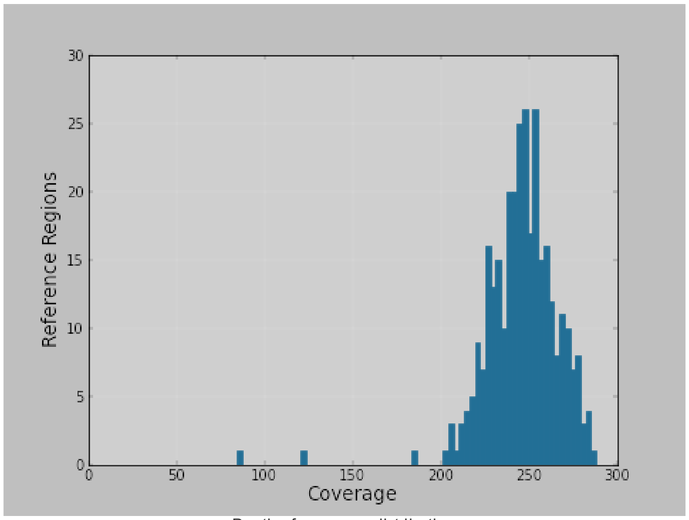

Depth of coverage distribution

Mapping

Post-Filter Reads	169114	Mapped Polymerase Read Length 95%	8413 bp
Mapped Read Length of Insert	3036 bp	Mapped Polymerase Read Length Max	14988 bp

	Mapped Reads	Mapped Polymerase Read Length	Mapped Subreads	Mapped Subread Bases	Mapped Subread Length	Mapped Subread Accuracy
All Movies	121138	3580 bp	153737	419021860 bp	2725 bp	86.31%
c100436120630000001523043602011381_s1_p0	15046	3714 bp	19323	54055836	2797 bp	86.01%
c100436120630000001523043602011382_s1_p0	16573	3400 bp	20567	54786803	2663 bp	86.82%
c100436120630000001523043602011383_s1_p0	19885	3577 bp	25209	68598621	2721 bp	86.15%
c100436120630000001523043602011384_s1_p0	20250	3469 bp	25281	68193099	2697 bp	86.57%
c100432341270000001523045902011320_s1_p0	17288	3483 bp	21673	57800942	2666 bp	86.28%
c100436120630000001523043602011385_s1_p0	16228	3885 bp	21493	60767967	2827 bp	86.34%
c100432341270000001523045902011321_s1_p0	15868	3579 bp	20191	54818592	2715 bp	85.99%

Mapped Subread Accuracy

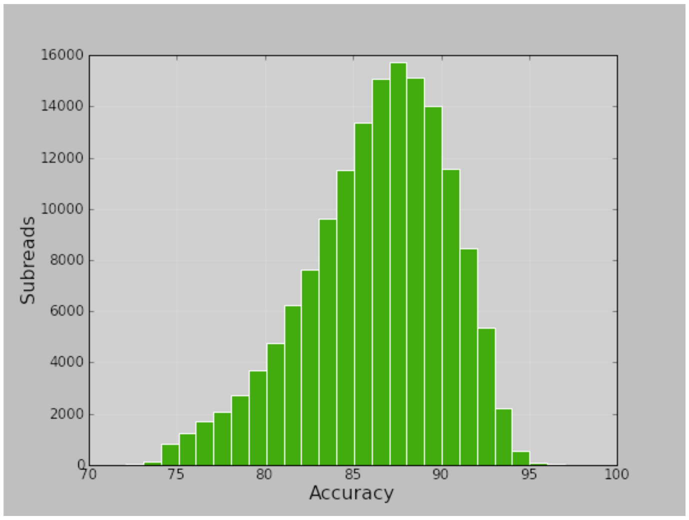

Mapped Subread Length

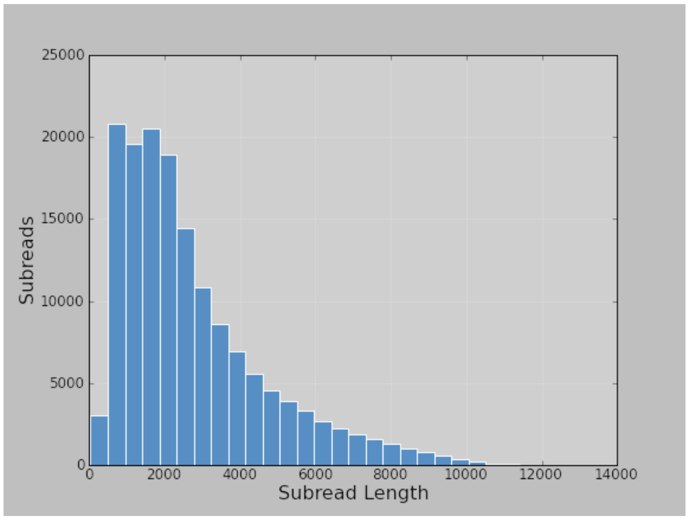

Mapped Polymerase Read Length

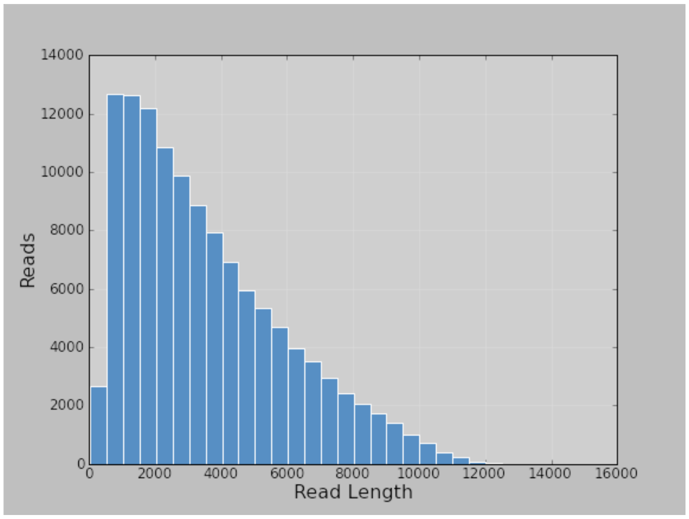

Generated by SMRT® Portal. Tue Jun 04 21:13:21 PDT 2013
For Research Use Only. Not for use in diagnostic procedures.
