# Supplementary material for: Comparing the genomes of Helicobacter pylori clinical strain UM032 and Mice-adapted derivatives
Source: Gut Pathog. 2013 Aug 19;5:25. doi: 10.1186/1757-4749-5-25 (PMC3751790; doi:10.1186/1757-4749-5-25)
Supplement: Additional file 3 — Assembly report for 299. [file 1757-4749-5-25-S3.pdf]

Reports for Job HP299

SMRT Cells: 8 Movies: 8

Overview

Job Metric	Value
Adapter Dimers (%)	8.85
Short Inserts (%)	1.44
Post-Filter Polymerase Read Bases	747846180
Post-Filter Polymerase Reads	204169
Post-Filter Polymerase Read Length	3663
Post-Filter Polymerase Read Quality	0.841
Max Contig Length	1601149
N50 Contig Length	1601149
Mapped Reads	158509
Mapped Read Length of Insert	2355
Reference Length - scf7180000000002	1601133
Bases Called (%) - scf7180000000002	100.00
Consensus Concordance - scf7180000000002	99.9979
Coverage - scf7180000000002	299.16

Adapters

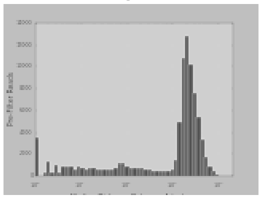

Observed Insert Length Distribution Histogram

Subread Filtering

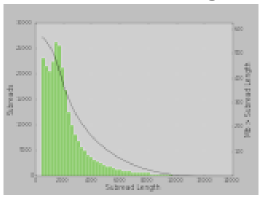

Subread Filtering

Coverage

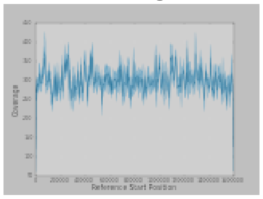

Coverage Across Reference

Coverage

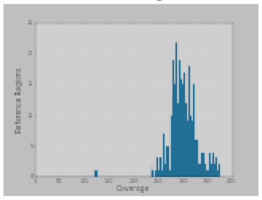

Depth of Coverage

Mapping

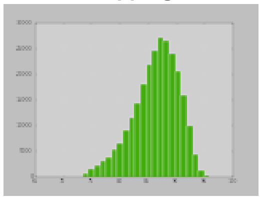

Mapped Subread Accuracy

Mapping

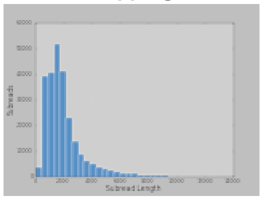

Mapped Subread Length

Mapping

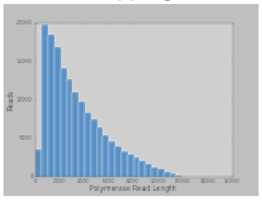

Mapped Polymerase Read Length

Corrections

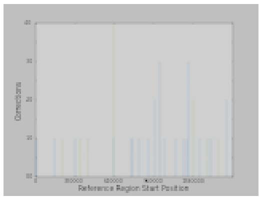

Corrections Across Contigs

Filtering

	Pre-Filter	Post-Filter
Polymerase Read Bases	1148521814 bp	747846180 bp
Polymerase Reads	601224	204169
Polymerase Read Length	1910 bp	3663 bp
Polymerase Read Quality	0.436	0.841

Polymerase Read Length

Post-Filter

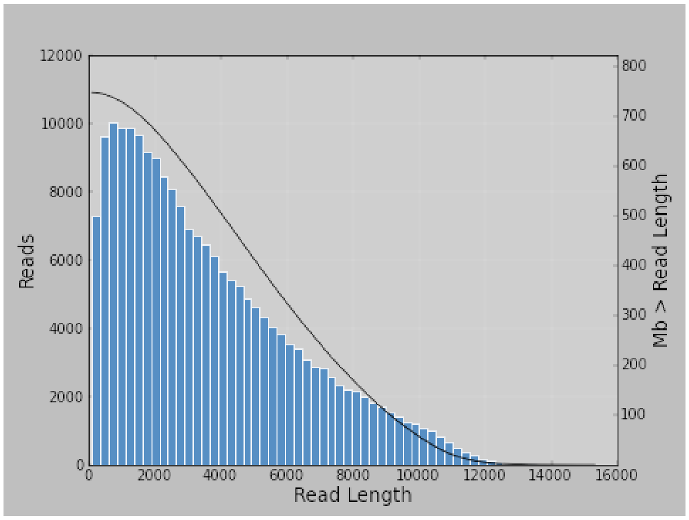

Polymerase Read Quality

Post-Filter

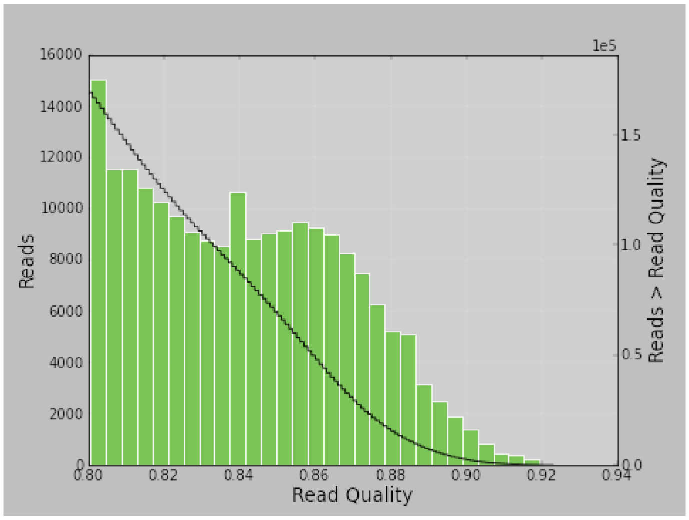

Subread Filtering

Subread Filtering

Post-Filter

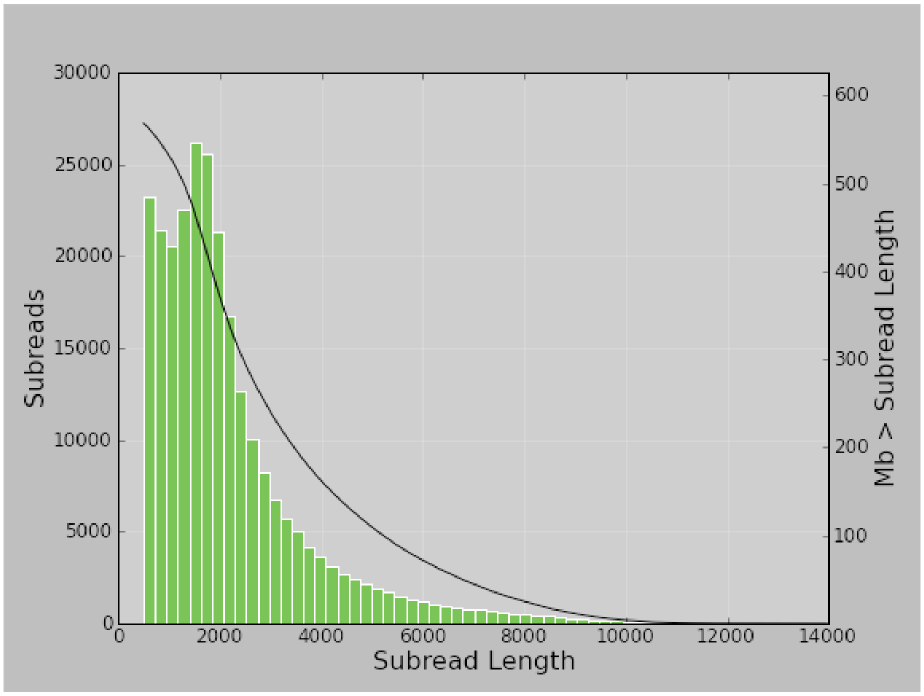

Adapters

Adapter Dimers (%)	8.85 %
Short Inserts (%)	1.44 %

Observed Insert Length Distribution Histogram

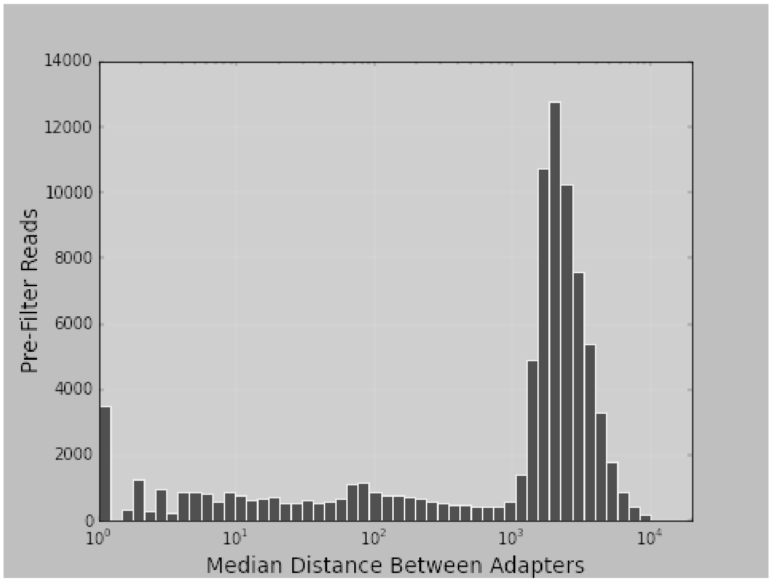

Loading

SMRT Cell ID	Productive ZMWs	Productivity 0 (%)	Productivity 1 (%)	Productivity 2 (%)
m120901_181316_42142_c100432341270000001523045902011322	75153	2.5	50.8	46.7
m120901_214856_42142_c100432341270000001523045902011324	75153	4.1	47.6	48.3
m120902_012436_42142_c100432341270000001523045902011326	75153	2.5	52.0	45.5
m120902_064806_42142_c100420481270000001523039112191272	75153	2.0	58.8	39.2
m120901_200106_42142_c100432341270000001523045902011323	75153	3.1	49.2	47.8
m120902_050016_42142_c100420481270000001523039112191271	75153	1.1	60.0	38.9
m120901_233646_42142_c100432341270000001523045902011325	75153	20.0	34.5	45.6
m120902_031226_42142_c100420481270000001523039112191270	75153	1.6	58.2	40.2

Pre-Assembly

Polymerase Read Bases	569190248	Length Cutoff	6738
Seed Bases	51021776	Pre-Assembled Bases	38023114
Pre-Assembled Yield	.745	Pre-Assembled Reads	6149
Pre-Assembled Read Length	6183	Pre-Assembled N50	6945

Top Corrections

Sequence	Position	Correction	Type	Coverage	Confidence	Genotype
scf7180000000002	325760	325760_325761insC	INS	100	50	haploid
scf7180000000002	1280415	1280415delTA	DEL	100	50	haploid
scf7180000000002	1545132	1545132_1545133insC	INS	100	50	haploid
scf7180000000002	17739	17739_17740insG	INS	100	49	haploid
scf7180000000002	638335	638335_638336insC	INS	100	49	haploid
scf7180000000002	783727	783727_783728insG	INS	100	49	haploid
scf7180000000002	787283	787283_787284insC	INS	100	49	haploid
scf7180000000002	912647	912647_912648insG	INS	100	49	haploid
scf7180000000002	1000864	1000864_1000865insG	INS	100	49	haploid
scf7180000000002	1228588	1228588_1228589insC	INS	100	49	haploid
scf7180000000002	1243928	1243928_1243929insC	INS	100	49	haploid
scf7180000000002	1394917	1394917delA	DEL	100	49	haploid
scf7180000000002	1548864	1548864_1548865insG	INS	100	49	haploid
scf7180000000002	157165	157165_157166insG	INS	100	48	haploid
scf7180000000002	223405	223405delT	DEL	100	48	haploid
scf7180000000002	362791	362791delA	DEL	100	48	haploid
scf7180000000002	429019	429019delT	DEL	100	48	haploid
scf7180000000002	631913	631913delTAAA	DEL	100	48	haploid
scf7180000000002	844746	844746_844747insC	INS	100	48	haploid
scf7180000000002	973436	973436_973437insGG	INS	100	48	haploid
scf7180000000002	1003913	1003913_1003914insGG	INS	100	48	haploid
scf7180000000002	1043382	1043382_1043383insC	INS	100	48	haploid
scf7180000000002	1134743	1134743_1134744insC	INS	100	48	haploid
scf7180000000002	1199638	1199638_1199639insG	INS	100	48	haploid
scf7180000000002	1243110	1243110_1243111insCC	INS	100	48	haploid
scf7180000000002	1336586	1336586_1336587insC	INS	100	48	haploid
scf7180000000002	1427587	1427587_1427588insG	INS	100	48	haploid
scf7180000000002	1494763	1494763delA	DEL	100	48	haploid
scf7180000000002	1610	1610_1611insC	INS	24	46	haploid
scf7180000000002	1599753	1599753_1599754insA	INS	17	45	haploid

Polished Assembly

Polished Contigs	1	Max Contig Length	1601149
N50 Contig Length	1601149	Sum of Contig Lengths	1601149

Corrections

Reference	Reference Length	Bases Called (%)	Consensus Concordance	Coverage
scf7180000000002	1601133	100.00	99.9979	299.16

Corrections Across Contigs

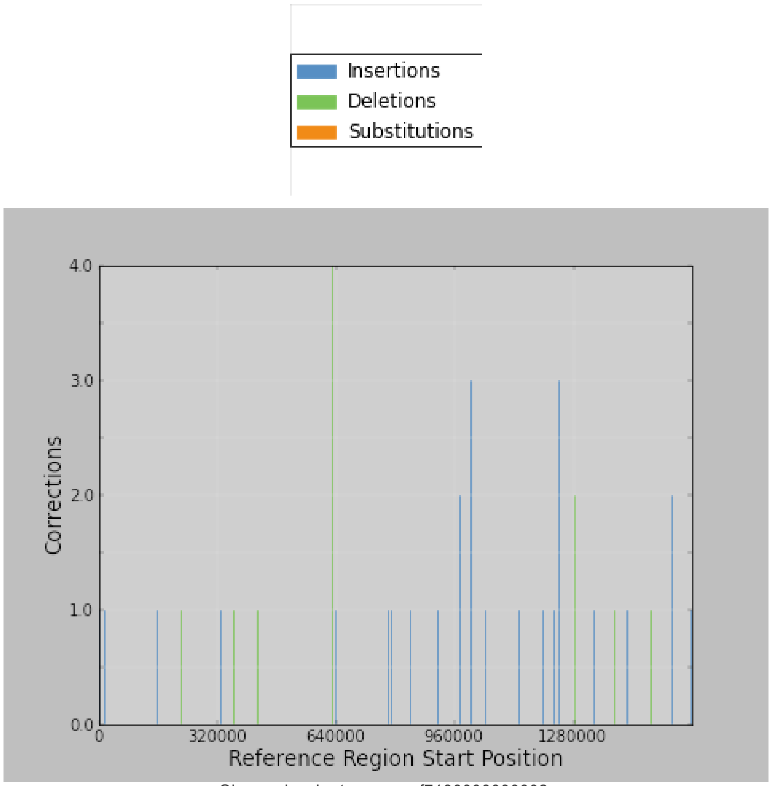

Observed variants across scf7180000000002.

Coverage

Coverage	299.16
Missing Bases (%)	0.00

Coverage Across Reference

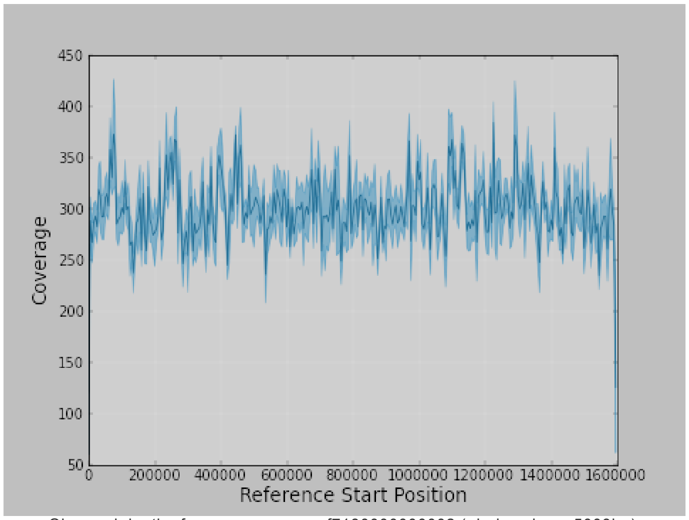

Observed depth of coverage across scf7180000000002 (window size = 5003bp).

Depth Of Coverage

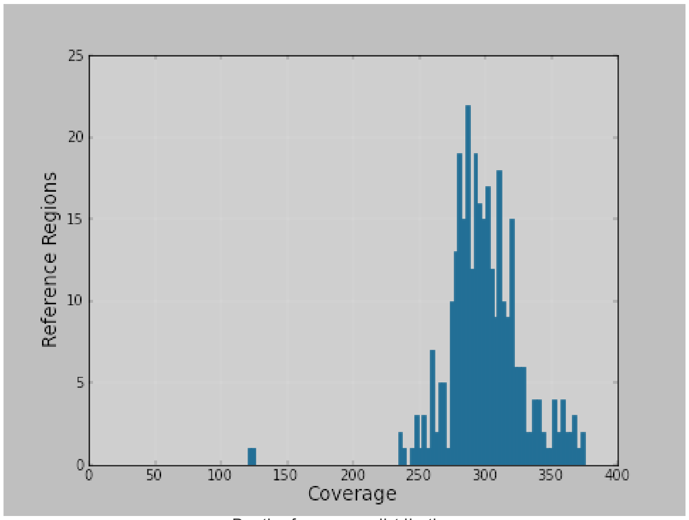

Depth of coverage distribution

Mapping

Post-Filter Reads	204169	Mapped Polymerase Read Length 95%	8285 bp
Mapped Read Length of Insert	2355 bp	Mapped Polymerase Read Length Max	14857 bp

	Mapped Reads	Mapped Polymerase Read Length	Mapped Subreads	Mapped Subread Bases	Mapped Subread Length	Mapped Subread Accuracy
All Movies	158509	3378 bp	252635	507646256 bp	2009 bp	86.35%
c100432341270000001523045902011322_s1_p0	18399	3427 bp	29640	59410569	2004 bp	86.04%
c100432341270000001523045902011325_s1_p0	10667	3185 bp	16715	32605139	1950 bp	87.13%
c100432341270000001523045902011323_s1_p0	19188	3400 bp	30755	61885626	2012 bp	86.45%
c100432341270000001523045902011324_s1_p0	18102	3453 bp	29619	59353511	2003 bp	86.37%
c100432341270000001523045902011326_s1_p0	21349	3250 bp	33203	65723477	1979 bp	86.43%
c100420481270000001523039112191271_s1_p0	24269	3389 bp	38499	77831880	2021 bp	86.20%
c100420481270000001523039112191272_s1_p0	21930	3502 bp	35554	72520857	2039 bp	86.15%
c100420481270000001523039112191270_s1_p0	24605	3343 bp	38650	78315197	2026 bp	86.43%

Mapped Subread Accuracy

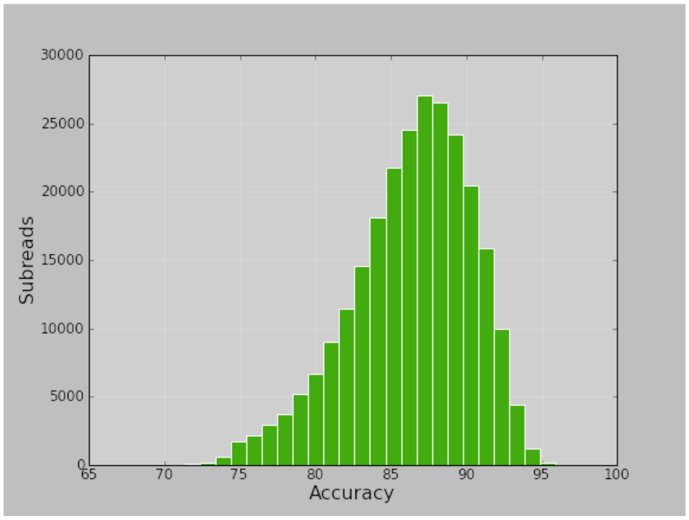

Mapped Subread Length

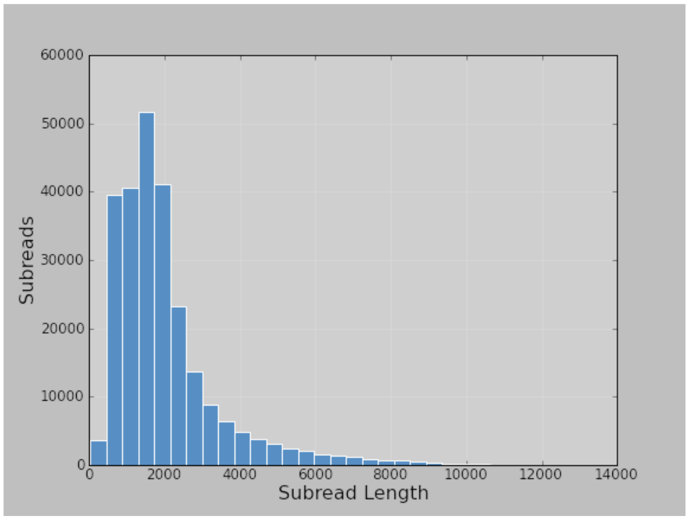

Mapped Polymerase Read Length

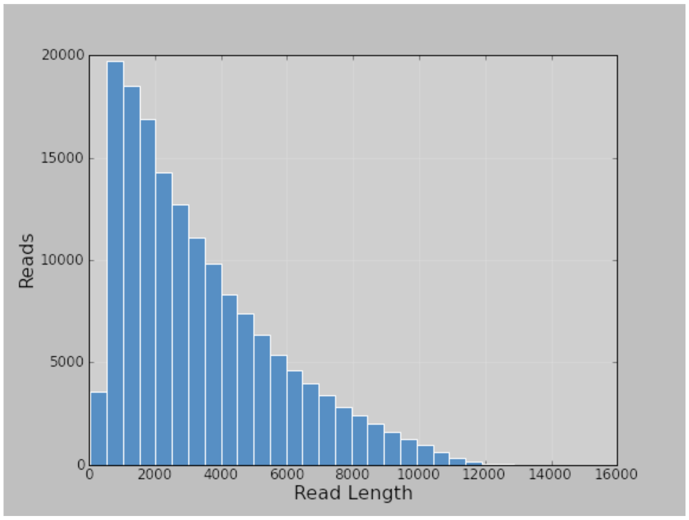

Generated by SMRT® Portal. Tue Jun 04 21:10:44 PDT 2013
For Research Use Only. Not for use in diagnostic procedures.
